# Diversity in genomic organisation, developmental regulation and distribution of the murine PR72/B" subunits of protein phosphatase 2A

**DOI:** 10.1186/1471-2164-9-393

**Published:** 2008-08-20

**Authors:** Karen Zwaenepoel, Justin V Louis, Jozef Goris, Veerle Janssens

**Affiliations:** 1 Protein Phosphorylation and Proteomics Group, Dept. Molecular Cell Biology, Faculty of Medicine, K.U. Leuven, Gasthuisberg O&N1, Herestraat 49 box 901, B-3000 Leuven, Belgium

## Abstract

**Background:**

Protein phosphatase 2A (PP2A) is a serine/threonine-specific phosphatase displaying vital functions in growth and development through its role in various signalling pathways. PP2A holoenzymes comprise a core dimer composed of a catalytic C and a structural A subunit, which can associate with a variable B-type subunit. The importance of the B-type subunits for PP2A regulation cannot be overestimated as they determine holoenzyme localisation, activity and substrate specificity. Three B-type subunit families have been identified: PR55/B, PR61/B' and PR72/B", of which the latter is currently the least characterised.

**Results:**

We deduced the sequences and genomic organisation of the different murine PR72/B" isoforms: three genes encode nine isoforms, five of which are abundantly expressed and give rise to genuine PP2A subunits. Thereby, one novel subunit was identified. Using Northern blotting, we examined the tissue-specific and developmental expression of these subunits. All subunits are highly expressed in heart, suggesting an important cardiac function. Immunohistochemical analysis revealed a striated expression pattern of PR72 and PR130 in heart and skeletal muscle, but not in bladder smooth muscle. The subcellular localisation and cell cycle regulatory ability of several PR72/B" isoforms were determined, demonstrating differences as well as similarities.

**Conclusion:**

In contrast to PR55/B and PR61/B', the PR72/B" family seems evolutionary more divergent, as only two of the murine genes have a human orthologue. We have integrated these results in a more consistent nomenclature of both human and murine PR72/B" genes and their transcripts/proteins. Our results provide a platform for the future generation of PR72/B" knockout mice.

## Background

Protein phosphatase 2A (PP2A), one of the major serine/threonine protein phosphatases in the cell, is involved in the control of a large number of cellular events including cell growth, intracellular signalling, DNA replication, transcription, translation, cell differentiation and cell transformation [1,2]. The key to understand how PP2A is capable of regulating such diverse, and sometimes even opposite functions, is its structure. The core of PP2A consists of a structural PR65/A subunit and a catalytic C subunit, both existing in two isoforms, α and β. To this PP2A dimer (PP2A_D_), a third regulatory B-type subunit can bind. It is generally believed that the regulatory B-type subunits target the phosphatase to distinct substrates and intracellular localisations. At present approximately 20 regulatory B-type subunits have been described. Based on their primary structure, they can be divided into three families: PR55/B, PR61/B' (also called B56) and PR72/B" [1]. They share two conserved A subunit binding domains (ASBD) [3]. In theory, about 80 different combinations of trimeric ABC holoenzymes can be formed. How many actually exist in the cell, is unknown and most probably differs in different tissues due to the tissue-specific expression of some PP2A subunits [1]. Furthermore, phosphorylation and methylation of the catalytic C subunit play an important role in the assembly of specific trimeric holoenzymes [4,5].

In the present study, we focus on the regulatory PR72/B" subunit family named after the molecular weight of the first identified member [6]. In mammals, sofar 6 members have been described: PR72 [6], PR130 [6], PR70 [7], PPP2R3L product [8], G5PR [9] and mPR59 [10], all sharing a conserved region with two ASBDs important for binding to PP2A_D_. Characteristically for this family, are – in addition to both ASBDs – two Ca^2+^-binding EF-hand motifs [11]. Mutation analysis of these EF-hand motifs together with several binding and activity studies indicate that Ca^2+ ^can influence the heterotrimeric assembly and catalytic activity of the B"-containing PP2A [11-14].

PR72 and PR130, the founding members of the B" family, are two N-terminal splice variants with a different tissue distribution pattern. PR72 is highly abundant in heart and skeletal muscle and barely detectable in other tissues. PR130, on the other hand, has a more widespread distribution [6]. Both splice variants have a role in Wnt signalling since they both regulate Naked Cuticle (Nkd) function, yet apparently in opposite ways [15,16]. Furthermore, addition of IQ-1, a compound which disrupts binding of PR72 and PR130 to both PP2A_D _and Nkd, results in prevention of embryonic stem cell differentiation due to a change of co-activators associating with β-catenin [17]. In addition, PR72-containing PP2A (PP2A_T72_) is also responsible for the glutamate-dependent dephosphorylation of Thr75 in dopamine- and cAMP-regulated phosphoprotein of 32 kDa (DARPP-32) in dopaminoceptive neuronal cells of the striatum [12]. PR130-containing PP2A (PP2A_T130_) has been described as an interacting protein of CG-NAP (centrosome and Golgi localised PKN-associated protein), a scaffolding protein that assembles several protein kinases (PKA, PKN) and protein phosphatases (PP1, PP2A_T130_) on centrosome and Golgi apparatus [18]. PP2A_T130 _is also suggested to be involved in the calcium release from the sarcoplasmic reticulum of heart cells as it can interact with the ryanodine receptor type 2, a heart-specific Ca^2+ ^channel found to be hyperphosphorylated in some patients with heart failure [19]. In *Xenopus laevis*, an additional splice variant, named XN73, has been found. This protein contains the specific N-terminus of PR130 followed by a short tail of 7 amino acids and thus lacks the ASBD necessary for PP2A_D_-binding. Consequently, this protein is not a regulatory PR72/B" subunit *strictu senso *[7] but-based on its sequence-might compete in binding to other cellular partners of PR130.

After identification of PR48 as a B" subunit family member which can bind Cdc6 [20] and therefore involved in regulation of the cell cycle, it was found that PR48 represents a partial clone of a larger human B" subunit, PR70 [7]. Currently, two PR48-containing isoforms have been described with different N-termini: PR70 and PPP2R3L product. PPP2R3L product is mainly expressed in heart and skeletal muscle [8], whereas PR70 is ubiquitously expressed [7]. Recently, various links between PR70 and cell cycle progression have been discovered. PR70 can bind retinoblastoma protein (pRb), thereby regulating its phosphorylation status following oxidative stress [13] and it can associate with Cdc6 [14].

Another member of the B" subunit family is named G5PR. It has a wide expression pattern and can bind both PP2A_D _and PP5 [9]. Furthermore, G5PR can interact with GANP, a DNA-primase which is selectively up-regulated in germinal centre B cells after immunisation with T cell-dependent antigens [9]. B-cell-specific G5PR knockout mice display a decreased number of splenic B cells. This enhanced cell death, specifically induced upon antigen binding to the specific B cell receptor, is caused by an increased activation of c-Jun NH_2_-terminal protein kinase and Bim [21]. In parallel, T-cell-specific G5PR knockout mice display a decreased number of thymocytes. The enhanced cell death, mainly seen in CD4 and CD8 double positive thymocytes, is accompanied by increased activation of both c-Jun NH2-terminal protein kinase and caspase-3, but not Bim [22].

mPR59 is a mouse-specific B" subunit, discovered as an interacting protein of p107, a Rb related protein [10]. No human orthologue of mPR59 is found so far. It is expressed in various tissues and overexpression can regulate p107 phosphorylation, causing an increase of cells in the G_1 _phase of the cell cycle [10]. Furthermore, mPR59 co-purifies with the L-Type Calcium Channel Ca_v_1.2, a voltage-gated Ca^2+ ^channel important for Ca^2+ ^influx in cells of the cardiovascular system, heart and brain [23].

Undoubtedly, the generation of (additional) PR72/B" knockout mice will be a valuable tool to obtain further insights into the potential physiological roles of the PR72/B" regulatory subunits. To provide a framework for the generation and analysis of such PR72/B" knockout mice, we present a comprehensive overview of the murine PR72/B"-encoding genes, their exon/intron organisation, their (alternative) transcripts and their developmental and tissue-specific expression. Surprisingly and in contrast to the murine PR55/B [24] and PR61/B' [25] families of PP2A subunits, the murine PR72/B" subunits have evolved somewhat differently compared to their human orthologues. As a consequence the current PR72/B" nomenclature is confusing, and we now propose some changes to make it more consistent. At the cellular level, we determine the subcellular localisation of the major PR72/B" subunits and their ability to affect cell cycle progression upon overexpression. Evidence is presented for at least one novel murine PR72/B" isoform, adding to the high diversity of PP2A B-type subunits.

## Results

### The murine B" family isoforms

In mammals, four genes of the B" regulatory subunit family of PP2A have been described, giving rise to six isoforms: PR72 [6], PR130 [6], PR70 [7], PPP2R3L product [8], G5PR [9] and mPR59, an isoform sofar only found in mice [10].

Upon searching the NCBI database for murine B" family members via the BLAST algorithm, we retrieved the complete protein sequence of two murine PR72 isoforms: the human PR72 orthologue [GenBank:BAE21013] (referred to as mPR72/B"α2) and a shorter variant missing the last 41 residues [GenBank:BAC28935] (referred to as mPR72/B"α4). In addition, one mG5PR isoform [GenBank:NP_067504] (referred to as mG5PR/B"γ) and four mPR59 isoforms were retrieved: mPR59 as described by Voorhoeve *et al. *[GenBank:AAC98973] (referred to as mPR59/B"δ2) [10], two mPR59 variants containing different N-termini [GenBank:AAH59852 and BAE25309] (referred to as mPR59/B"δ1 and mPR59/B"δ3 respectively) and a mPR59/B"δ3 variant harbouring an alternative C-terminus [GenBank:AAH96544] (referred to as mPR59/B"δ4). Additionally, two partial protein sequences of murine PR130 homologues were retrieved, one containing the first 615 amino acids of the PR130 specific N-terminus [GenBank:BAC31413] and the other containing the last 203 amino acids of the specific N-terminus followed by 12 additional residues [GenBank:BAC37349], resembling *Xenopus *XN73 (referred to as mPR130/B"α3). Of the full-length mPR130 (referred to as mPR130/B"α1), only a predicted sequence [GenBank:XP_135153] could be found.

To confirm these *in silico *data and to get an idea about the abundancy of these clones, we scanned the NCBI database for murine EST-clones containing the B"-specific N- or C-termini. Only very few EST-clones were found carrying the sequence of the mPR72 C-terminus lacking 41 residues [GenBank:BE852268 and CA984544], the XN73-like truncated mPR130 C-terminus [GenBank:AV356684] and the alternative mPR59 C-terminus [GenBank:BB357167], making it rather unlikely that murine B" family members containing these termini (mPR130/B"α3, mPR72/B"α4 and mPR59/B"δ4) do abundantly exist. In contrast, five EST-clones of the specific N-terminus of mPR59/B"δ3 and multiple (> 15) EST-clones of the other B"-specific N- and C-termini were found, suggesting that mPR72/B"α2, mPR130/B"α1, mG5PR/B"γ, mPR59/B"δ1, PR59/B"δ2, and PR59/B"δ3 are much more abundant, and probably represent the main murine B" isoforms.

**Figure 1:**
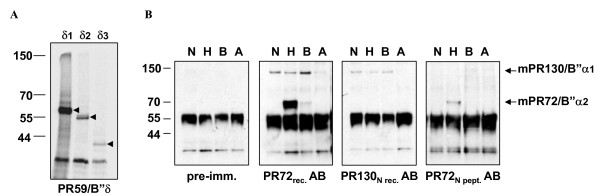
**Diversity of the murine PR130/72 and PR59 isoforms**. **A: **Autoradiography of [^35^S]-labelled *in vitro *transcription-translation products of plasmids (1 μg) containing cDNA of murine PR59/B"δ1 (lane δ1), PR59/B"δ2 (lane δ2) and PR59/B"δ3 (lane δ3). **B: **Immunoprecipitation of mPR72/PR130 isoforms from different murine cells and tissues (N = NIH 3T3, H = heart, B = brain, A = adrenal gland) using pre-immunisation sera (as negative control) and three different PR72/PR130 antibodies: PR72_rec. _AB recognizes all putative PR72 isoforms and the PR130 isoforms which contain the common C-terminal region, PR130_N rec. _AB recognizes all PR130 isoforms, and PR72_N pept. _AB recognizes all PR72 isoforms.

Since mPR59/B"δ1 and mPR59/B"δ3 are putatively novel, and to further confirm their existence, we performed a reverse transcription on NIH 3T3 and murine heart RNA using primer E_3 _annealing in the mPR59 3' UTR. With the resulting cDNAs as templates and the use of five different primers, annealing with the specific N-termini of PR59/B"δ1–4 (B_δ1_, B_δ2_, B_δ3/4_) and/or the specific C-termini of PR59/B"δ1–3 (E_2_) and PR59/B"δ4 (E_1_), we were able to generate several DNA fragments, which after sequencing, were shown to correspond to PR59/B"δ1, PR59/B"δ2 and PR59/B"δ3. *In vitro *transcription-translation reactions of these cDNAs revealed proteins of about 60 kDa (PR59/B"δ1), 55 kDa (PR59/B"δ2) and 40 kDa (PR59/B"δ3) (Figure 1A), implying that not only the messengers of PR59/B"δ1,δ2,δ3 are present in murine cells, but that they can also be translated into proteins. A cDNA for mPR59/B"δ4 could not be amplified using this approach, confirming its low occurrence in the EST database.

A similar approach was undertaken to confirm the existence of the four mPR130/PR72 isoforms. In this case RT-PCR of NIH 3T3 or murine heart RNA led to the amplification of mPR130/B"α1 and mPR72/B"α2 cDNA, but not of mPR130/B"α3 or mPR72/"α4. To confirm this at the protein level, we prepared protein extracts of murine embryonic fibroblast NIH 3T3 cells and several murine adult tissues: heart, in which both human PR130 as PR72 are highly expressed [6]; adrenal gland, the tissue from which the mPR130/B"α3 EST-clone [GenBank:AV356684] was isolated, and brain. Three antibodies were used to perform immunoprecipitations of the different mPR130/PR72 isoforms: a PR72 AB, raised against full-length recombinant human PR72 [11]; a PR130_N _AB, raised against the recombinant specific N-terminus of human PR130 (AA 1–664) and a peptide PR72 AB, raised against the first 19 amino acids of the human PR72-specific N-terminus. The results demonstrate immunoreactivity at 140 kDa and 70 kDa, corresponding to mPR130/B"α1 and mPR72/B"α2 (Figure 1B). No other specific bands could be observed, even upon prolonged exposure, confirming our *in silico *and RT-PCR analysis. Consequently, it can be concluded that should the mPR130/B"α3 and/or mPR72/B"α4 isoforms exist, their expression would be very low or highly restricted in time and/or space.

**Figure 2:**
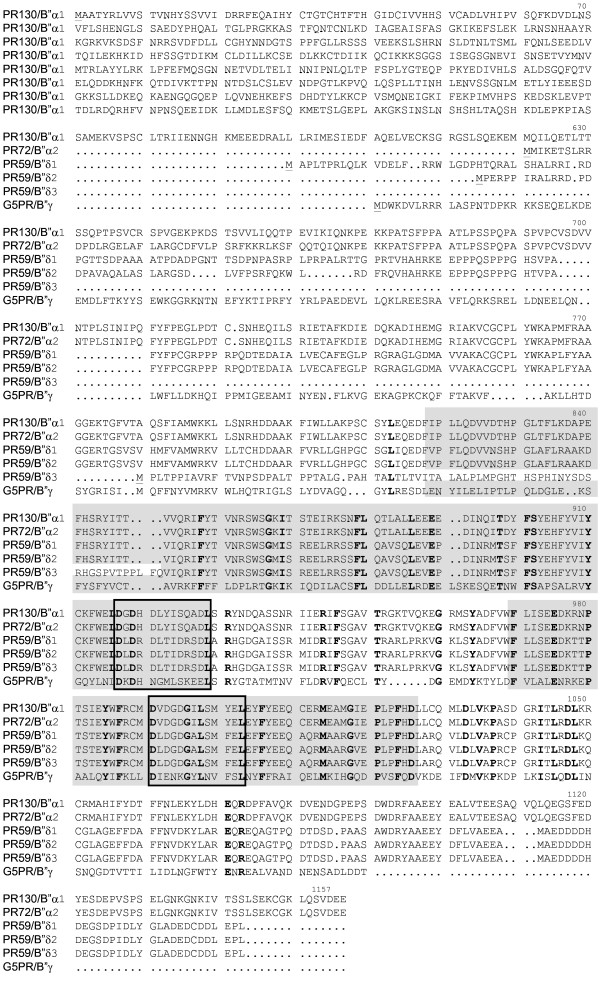
**Alignment of the amino acid sequences of the murine B" isoforms**. Residues identical for all isoforms are shown in bold. The two ASBDs are marked in grey shade and the boxes represent the two conserved EF-hand motifs. For each isoform, the start Methionine is underlined. The first 8 lines represent only the specific sequences of the PR130 N-terminus.

Together, six main B" family members are present in mice: mPR130/B"α1, mPR72/B"α2, mG5PR/B"γ, mPR59/B"δ1, mPR59/B"δ2 and mPR59/B"δ3, with mPR59/B"δ1 and δ3 being novel ones. A protein alignment of these murine B" isoforms (Figure 2) reveals that they all share a conserved region, harbouring both ASBD and both EF-hand motifs, with the exception of mPR59/B"δ3 which misses 31 amino acids of the first ASBD. They mainly differ within their N- and/or C-termini, which may be responsible for isoform-specific functions. Surprisingly, no evidence of murine PR70 and PPP2R3L product orthologues could be found. The murine proteins most closely resembling these isoforms are PR59/B"δ1 (58% identity, 66% similarity with hPR70) and PR59/B"δ2 (57% identity; 66% similarity with the PPP2R3L product).

### Genomic organisation of the three murine B" genes

**Figure 3:**
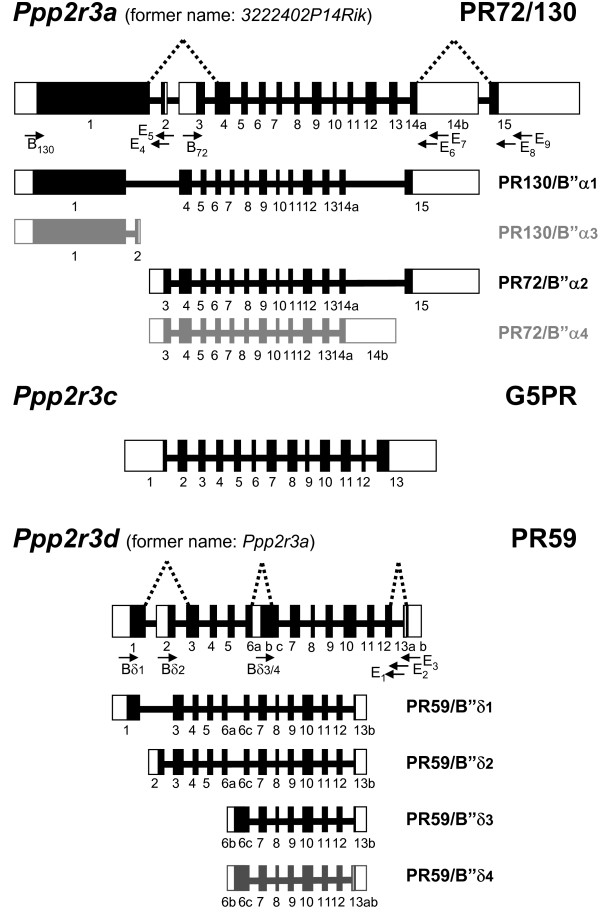
**Organisation of the murine B"-encoding genes and their transcripts**. The boxes represent the exons (on scale) and the lines represent the introns (not on scale). The coding regions (black boxes) and untranslated regions (white boxes) are indicated. Splicing events are denoted by dotted lines. Below the genomic structures, all putative transcripts are indicated: the main transcripts are coloured in black (at least 5 EST clones were retrieved), while less abundant transcripts are coloured in grey (less than 5 EST clones were found). Primers used for RT-PCR are also indicated (forward primers: B_72_, B_130_, B_δ1_, B_δ2 _and B_δ3/4_; reverse primers: E1 to E9).

An alignment of the nucleotide sequences of the murine PR72/B" isoforms suggests that all novel isoforms are the result of alternative splicing of the genes encoding previously described members. By BLASTN analysis of the murine genome using the murine cDNA sequences, we could solve the genomic organisation of these three murine B" genes (Figure 3). The intron-exon boundaries were deduced by comparing the sequences obtained from the genomic clones and the respective cDNAs. Almost all boundaries were found to follow Chambon's rule (GT-AG) for splice donor and acceptor sites [26] (Table 1).

**Table 1:** Exon-intron organisation of the murine PR72/B" genes.

***Ppp2r3a***	exon	exon size (bp)	5'-splice donor	intron size (bp)	3'-splice acceptor
	1	2434	ATCAAG**gt**aaga	4838	tctt**ag**ATTAGA
	2	98	AGGTCT**gt**aacc	7123	tccc**ag**AAGGCT
	3	441	ACACAG**gt**ttga	4205	ttcc**ag**ATTCAA
	4	267	GCAAAG**gt**aaca	8122	ttgc**ag**GTCTGT
	5	104	GAAAAA**gt**aagt	5495	ttgt**ag**GTTGCT
	6	103	CTTCAG**gt**gata	4932	tta**ag**GATGTG
	7	75	ACCACG**gt**agga	21453	aact**ag**GTTGTT
	8	87	TTGCAA**gt**atgc	5049	tttc**ag**ACTCTG
	9	157	ACCAGG**gt**aagt	3058	attt**ag**CTTCAT
	10	49	AACAAG**gt**atga	1074	cttc**ag**AGGAAA
	11	90	TACCAG**gt**aaag	17238	tcct**ag**CATTGA
	12	176	GTGATG**gt**aagg	469	tcat**ag**GCAGAA
	13	119	CAGAAG**gt**aaca	1179	aatc**ag**GATGTT
	14a	107	AGAAGG	/	**GT**GAGT
	14b	1174	GAGATA**gt**cctc	15879	ttcc**ag**CTCATT
	15	1575			

***Ppp2r3c***	exon	exon size (bp)	5'-splice donor	intron size (bp)	3'-splice acceptor

	1	866	CAAAGA**gt**gagt	3572	tact**ag**GGAAAA
	2	128	TACAGG**gt**aagt	378	ttta**ag**TTGCCA
	3	105	TTGCAG**gt**acga	520	ctta**ag**AACTTA
	4	113	GTGCAA**gt**aaga	3898	tttt**ag**GCAATT
	5	98	GAAAAG**gt**gatt	1207	tgtc**ag**TTTGGC
	6	71	GAATCA**gt**gagt	2711	tccc**ag**GACCTG
	7	133	GAACAG**gt**aaaa	913	ccca**ag**GGAAGA
	8	156	CTGGAG**gt**aaat	76	cttt**ag**CTAAGA
	9	76	TCTATG**gt**aggc	381	tgca**ag**GTCAGT
	10	137	GAAATG**gt**agtt	3006	ctgt**ag**GACTAT
	11	138	TTTAGG**gt**aagt	1803	atac**ag**GCCATA
	12	60	GTCAAG**gt**tatt	1108	ttat**ag**GATGAA
	13	982			

***Ppp2r3d***	exon	exon size (bp)	5'-splice donor	intron size (bp)	3'-splice acceptor

	1	567	CAGGACggtgag	358	gcac**ag**ACCCGA
	2	321	CGGCAG**gt**gggc	405	ccac**ag**GTCCAT
	3	198	GCCAAG**gt**atgt	298	cccc**ag**GCCTGT
	4	105	CGCAAGtgagtg	409	acag**ag**TCCTGC
	5	101	CTGCAG**gt**gggc	672	tggc**ag**GATGTG
	6a	75	ACCACA	/	**GT**GAGC
	6b	373	TTCC**AG**	/	**GT**GATT
	6c	87	CTGCAG**gt**gtgg	452	ctgc**ag**GCTGTG
	7	157	AGCGGG**gt**gagt	260	ccgc**ag**CCATCT
	8	49	CACCAG**gt**gagt	276	ccgc**ag**GGCGAG
	9	90	CACCAG**gt**gagg	402	ccac**ag**CACCGA
	10	176	GCCCCG**gt**gagc	220	cccc**ag**GCCGGA
	11	119	CCGCAG**gt**gggt	412	ccac**ag**GACACT
	12	113	CGAAGG**gt**taga	296	tcac**ag**GCCCTG
	13a	37	CCC**AG**	/	**GT**CCGA
	13b	256			

Nucleotides in uppercase represent exonic sequences while nucleotides in lowercase represent intronic sequences. Bold letters indicate the exon-intron boundaries based on the AT-GT rule.

The genomic organisation of the gene giving rise to the murine PR130 and PR72 isoforms (*3222402P14Rik*, MGI: 2442104) was deduced from clone RP24-308L17 [GenBank:AC120390], containing the entire gene. To retain the logic in the PR72/B" gene nomenclature, we propose to rename this gene into *Ppp2r3a*, analogous to the human *PPP2R3A *gene. The mPR130/PR72 gene is located on chromosome 9F1 (101 Mb). This region corresponds to human chromosome 3q22, where *PPP2R3A *is located. The mPR130/PR72 gene (Figure 3) spans approximately 106 kb and consists of 15 exons. In addition to the exons giving rise to mPR130/B"α1 and mPR72/B"α2, we could also locate both the exon containing the sequence of the 12 specific C-terminal amino acids of mPR130/B"α3 (exon 2), and the exon responsible for the premature ending of PR72/B"α4 (exon 14B) at the correct sites within the gene. Since PR72/B"α2 is the result of an intra-exonic splicing event within exon 14, PR72/B"α4 might well be the result of a splicing error at this position, possibly explaining its low abundance. In the human PR130/PR72 gene (*PPP2R3A*), the exon responsible for the putative generation of a human PR72/B"α4 isoform is also present. The NCBI human EST database contains exactly one EST [GenBank:CA427549] containing this exon. In contrast, no sign of the specific C-terminal tail of either mPR130/B"α3 or XN73 could be found in *PPP2R3A*. In addition, no human EST-clones were retrieved containing the PR130-specific N-terminus followed by nucleotides other than those of the common part of PR130 and PR72.

**Figure 4:**
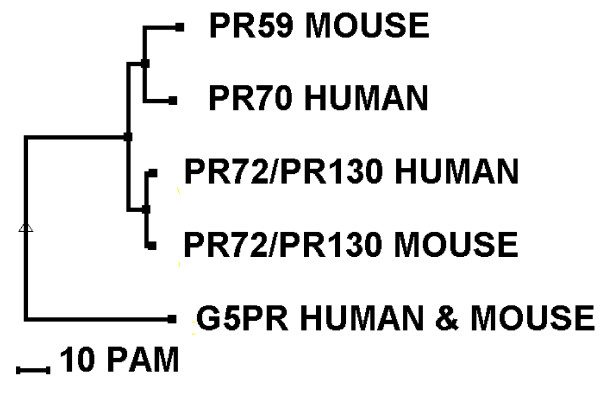
**Phylogenetic tree (MULTALIN)**. Phylogenetic tree based on protein sequence of human and murine B" regulatory subunits (human and murine PR72/B"α2 [GenBank:NP_871626 and BAE21013], human and murine PR130/B''α1 [GenBank:Q06190 and XP_135153], murine PR59/B''δ1,2,3 [GenBank:AAH59852, AAC98973 and BAE25309], human PR70 [GenBank:DAA00385], human PPP2R3L product [GenBank:CAI41975], human and murine G5PR/B''γ [GenBank:BAA91308, NP_067504].

To unravel the genomic organisation of the murine PR59 gene (MGI: 1335093), we analysed NCBI contig NW_001030923. Currently, this gene's symbol is *Ppp2r3a*, but as one could easily mistake it for the murine orthologue of *PPP2R3A*, we propose to change its name into *Ppp2r3d *(see also further). This gene spans approximately 9 kb and consists of 13 exons. The exons encoding the complete sequences of all mPR59 isoforms were found at the appropriate locations (Figure 3). To find the human *Ppp2r3d *homologue, we scanned the human genome using the BLASTN program with the different mPR59 isoforms as input. The closest match was the human PR70/PPP2R3L product gene (*PPP2R3B/L*), followed by the human PR130/PR72 gene (*PPP2R3A*), confirming the similarity between PR70/PPP2R3L and mPR59 at the genomic level. Therefore, the murine *Ppp2r3d *and the human *PPP2R3B/L *gene may have evolved from a common ancestor gene. This hypothesis looks promising since to our knowledge no organism contains both a PR70/PPP2R3L product and a PR59-encoding gene. A phylogenetic tree, based on the protein sequences of various PR72/B" family members in man and mouse, further supports this hypothesis (Figure 4).

Analysis of the mG5PR genomic structure is based on NCBI contig NW_001030500, which is located on chromosome 12C2 (56 Mb, ENSEMBLE ContigView) and contains the entire gene (*Ppp2r3c*, MGI: 193009). The mG5PR gene (Figure 3) contains 13 exons and closely mimics the genomic organisation of the human gene [9].

### The mPR59/B"δ3 protein is not a genuine PP2Aregulatory subunit

**Figure 5:**
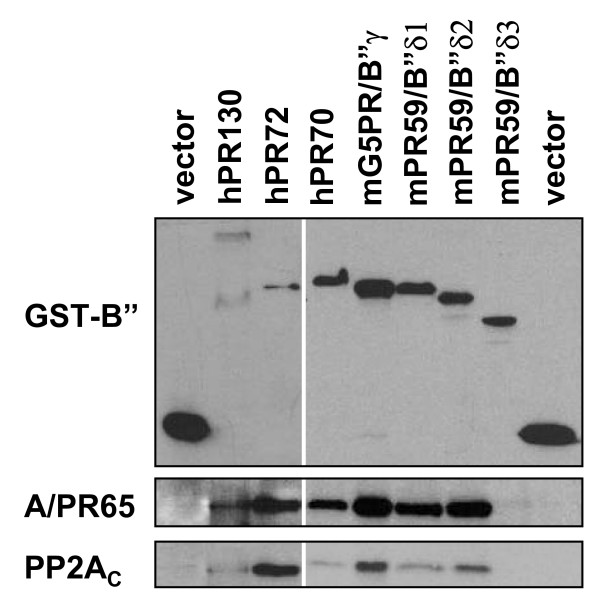
**Association of the main human and murine PR72/B" isoforms with PP2A_D_**. COS7 cells were transfected with GST, hPR130-GST, hPR72-GST, hPR70-GST, mG5PR/B"γ-GST, mPR59/B"δ1-GST, mPR59/B"δ2-GST and mPR59/B"δ3-GST. 48 h after transfection, a GST pull down assay was performed and binding of PR65/A and C subunits was evaluated via Western blotting using specific antibodies.

With the exception of XN73 and mPR130/B"α3, all B" family members contain a conserved region and specific N- and C-termini. The conserved region, which contains two ASBDs and two EF-hand motifs, is necessary for PR65/A binding [3]. Since the first ASBD is not intact in mPR59/B"δ3 (Figure 2), we wondered whether this isoform is still able to bind PP2A. To this end, we made GST-fusion proteins of the main B" family members and overexpressed these in COS-7 cells. After a GST-pull down assay, we evaluated the binding of both the PR65/A and catalytic subunit of PP2A via Western blotting. As expected, all main B" isoforms (hPR130, hPR72, hPR70, mG5PR/B"γ, mPR59/B"δ1 and mPR59/B"δ2) bind PP2A (Figure 5), and are therefore genuine B" subunits. In contrast, mPR59/B"δ3 fails to bind PP2A, suggesting it is not a regulatory subunit of PP2A (Figure 5). Like XN73 and mPR130/B"α3, it might be involved in regulation of PP2A by competing with the other mPR59 isoforms for binding to other binding partners.

### Tissue distribution of mPR130/B"α1, mPR72/B"α2, mPR59/B"δ1 and mPR59/B"δ2

**Figure 6:**
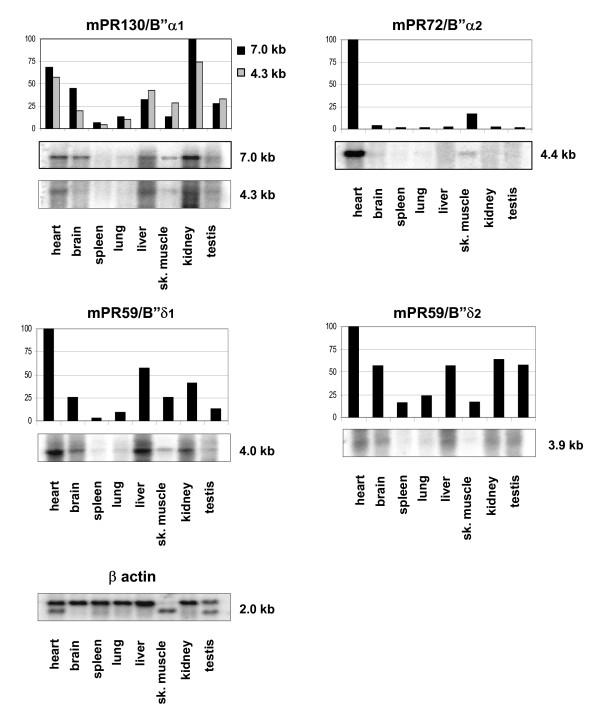
**Tissue distribution of the murine PR130/B"α1, PR72/B"α2, PR59/B"δ1/2 transcripts**. A first choice Mouse Blot I containing poly(A^+^) RNAs from ten different murine tissues (Clontech) was hybridised with isoform-specific RNA probes. For quantification we used the ImageQuant program from Molecular Dynamics. Percentages of expression are relative to the most intense band on each blot, which was given a value of 100%. Therefore only transcripts present on the same blot can be compared. A β-actin control hybridisation is shown at the bottom.

We determined the mRNA expression of the genuine murine B" regulatory subunits via Northern blot analysis (Figure 6). Using an antisense probe which hybridises to the first 330 nucleotides of mPR130, transcripts of 4.3 kb and 7.0 kb were detected. These transcripts are probably generated by the use of different polyadenylation sites. However, we can not exclude the possibility that the 4.3 kb transcript represents mPR130/B"α3. Both mPR130 transcripts have a similar expression pattern, being high in kidney and heart, intermediate in skeletal muscle, brain, liver and testis and low in lung and spleen. These findings are consistent with the previously published ubiquitous expression of human PR130 [6].

A probe specific for the N-terminal sequence of the mPR72 isoforms, detected a single band at 4.4 kb, likely corresponding to mPR72/B"α2. Similar to the expression pattern of PR72 in humans [6], mPR72/B"α2 is abundant in heart and skeletal muscle and barely detectable in other tissues (Figure 6).

As for mPR130/B"α1 and mPR72/B"α2, the transcripts of mPR59/B"δ1 and mPR59/B"δ2 were visualised using probes hybridising with their specific N-termini. The expression profiles obtained reveal a wide tissue distribution with some minor differences between the two splice variants: mPR59/B"δ1 expression is high in heart and liver, intermediate in kidney, brain and skeletal muscle and rather low in testis, lung and spleen, while mPR59/B"δ2 is highly abundant in heart, liver, brain, kidney and testis and less abundant in lung, skeletal muscle and spleen.

These results, together with the earlier observed broad mG5PR expression pattern [9] indicate a general high expression of all murine PP2A B" subunits in heart.

### Embryonic expression of the murine B" regulatory subunits

**Figure 7:**
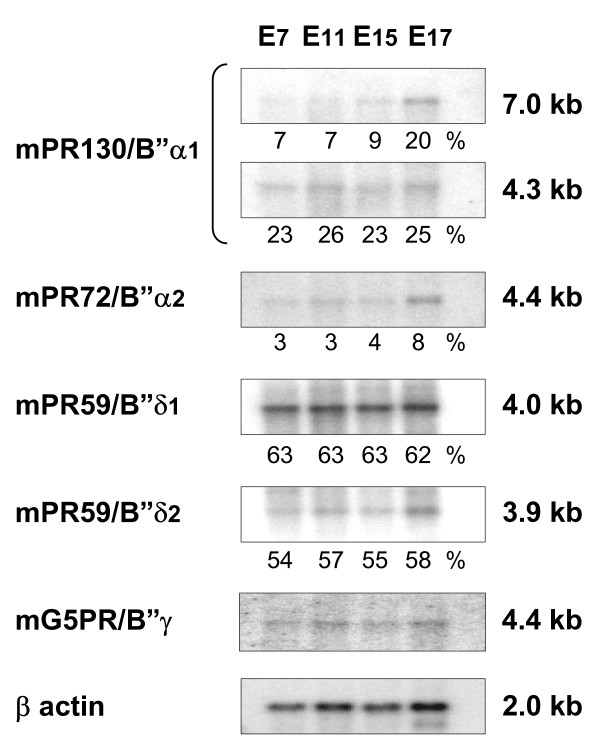
**Expression pattern of the main murine B" regulatory subunits in embryos**. A murine embryo multiple tissue Northern Blot containing poly(A^+^) RNAs from murine embryos at days 7, 11, 15 and 17 (Clontech) was hybridised with isoform-specific RNA probes. Embryonic expression of mPR130/B"α1, mPR72/B"α2, mPR59/B"δ1, mPR59/B"δ2 and mG5PR/B"γ is shown. For quantification we used the ImageQuant program from Molecular Dynamics. Percentages of expression are relative to the most intense band of the murine tissues blot (Figure 6). This way, a comparison with the relative expression of a specific isoform in the different tissues (Figure 6) can be made. The β-actin control hybridisation is shown at the bottom and was used to correct for equal loading.

In order to generate viable knockout mice, it can be important to establish whether a function of the protein of interest can be expected during embryonic development, as a general knockout of such a gene might lead to an embryonic lethal phenotype. Expression of the mPR130/B"α1, mPR72/B"α2, mPR59/B"δ1, mPR59/B"δ2 and mG5PR/B"γ subunits was examined via Northern blotting at embryonic days 7, 11, 15 and 17 (Figure 7). Hybridisation was performed using the same specific probes as for the tissue distribution determination. For mG5PR/B"γ, a probe spanning the first 450 nucleotides of mG5PR/B"γ was used. No additional splice variants, specific for embryonic stages, were observed for any of the main murine B" subunits. Expression of both mPR130/B"α1 and mPR72/B"α2 transcripts increases as embryonic development proceeds. This might suggest a role for these proteins in fetal growth and development. In previous studies, both hPR130 as well as hPR72 have been reported to influence Wnt signalling [15-17]. In contrast, the expression of mPR59/B"δ1, mPR59/B"δ2 and mG5PR/B"γ remains constant during all stages of embryonic development (Figure 7).

### Immunohistochemical analysis of mPR130/B"α1 and mPR72/B"α2 in different muscle tissues

All B" isoforms are highly expressed in heart. Moreover, mPR72/B"α2 expression is nearly restricted to muscle tissue. mPR130/B"α1, which has a broader expression pattern, is also substantially present in the different muscle tissues represented on the Northern blot. Since we have specific antibodies for both isoforms (Figure 1B), we examined the distribution of mPR130/B"α1 and mPR72/B"α2 in the different types of muscle tissue (heart, skeletal and smooth muscle) in more detail via immunohistochemistry.

**Figure 8:**
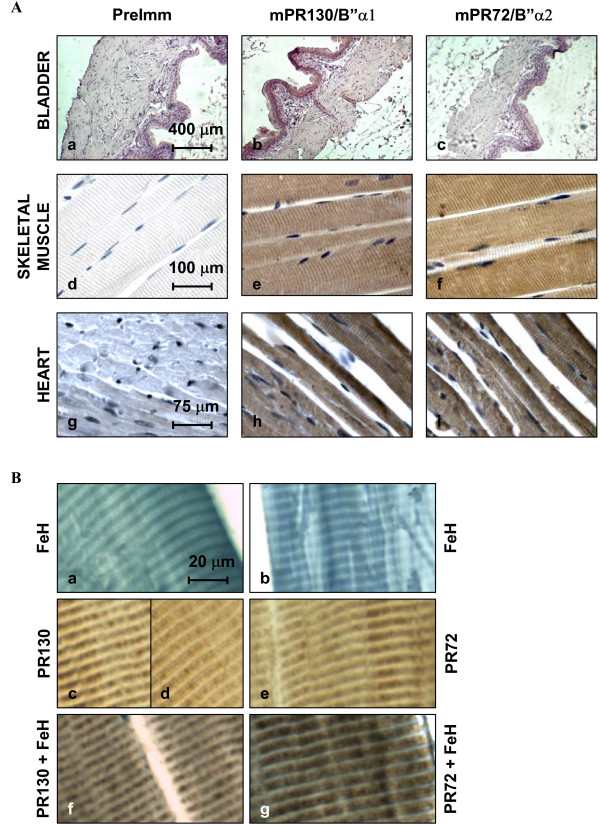
**Immunohistochemistry of mPR130/B"α1 and mPR72/B"α2 in muscle tissue (heart, skeletal muscle and smooth muscle)**. **A**: Sections of bladder (a-c), skeletal muscle (d-f) and heart (g-i) were costained with the nuclei-specific hematoxylin dye (blue) and antibodies specific for mPR130/B"α1 (b, e, h) or mPR72/B"α2 (c, f, i). As a control, sections were stained with pre-immune sera (a, d, g). **B: **Staining of longitudinal sections of skeletal muscle with iron hematoxylin (a, b), PR130-specific AB (c,d), PR72-specific AB (e) and costaining with iron hematoxylin and PR130 AB (f) or PR72 AB (g).

In longitudinal sections of skeletal muscle, mPR130/B"α1 and mPR72/B"α2 both intensively stain the muscle fibers in a striated pattern (Figure 8A, e–f). To evaluate whether these bands represent the I- or A-bands, we counterstained the sections with iron hematoxylin. This dye gives the A-band a dark blue colour and colocalises with mPR130/B"α1 and mPR72/B"α2 (Figure 8B, f–g). In one of the muscle fibers stained with mPR130/B"α1 AB, we could even observe a darker stained band positioned in the centre of the A-band (Figure 8B, d). This band probably represents the H-zone. In sections of murine myocardium, a similar striated staining pattern could be observed in the longitudinally positioned muscle fibers. Cross-sectional muscle fibers did not show staining of any specific structures (Figure 8A, h–i). In bladder, neither mPR130/B"α1 AB nor mPR72/B"α2 AB stained the smooth muscle fibers of the muscular layer. In contrast, mPR130/B"α1 staining could be seen in the transitional epithelial layer, lining the inner cavity of the bladder (Figure 8A, b–c). In none of the cases, specific staining of any structures was observed using pre-immune serum (Figure 8A, a,d,g).

In conclusion, PR130/B"α1 and PR72/B"α2 staining seems to be a specific feature of the A-band striations of heart and skeletal muscle, whereas staining is very low or absent in smooth muscle.

### Subcellular localisation of the murine B" subunits

**Figure 9:**
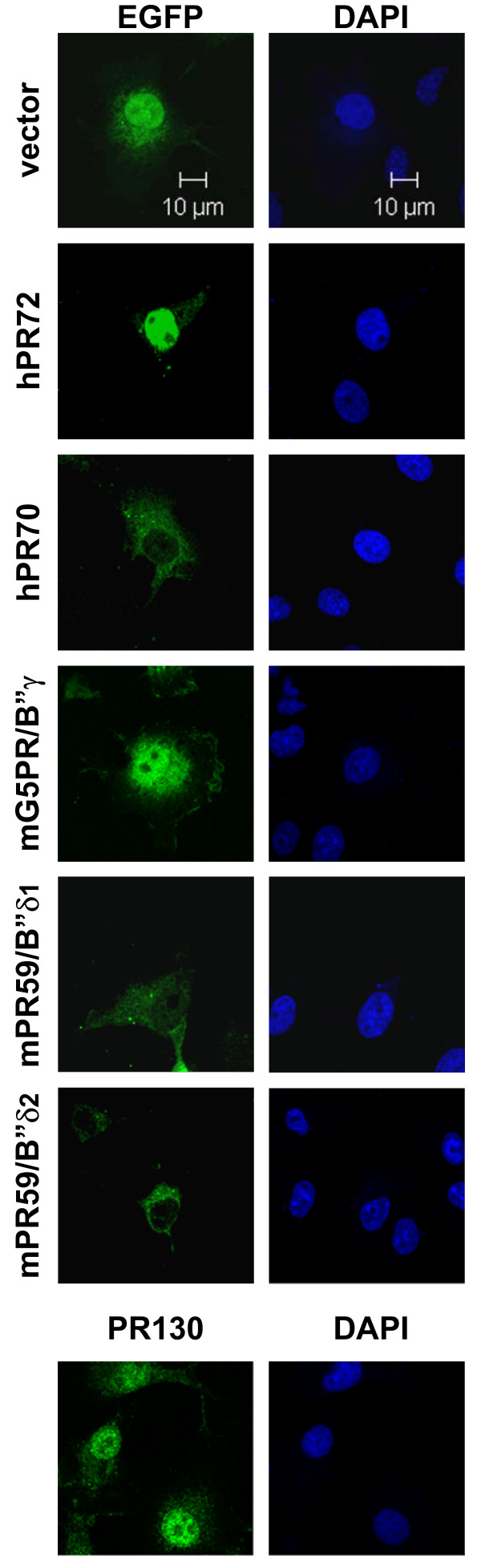
**Subcellular localisation of the main PR72/B" regulatory subunits in COS-7 cells**. Endogenous PR130 was visualised using PR130_N __rec_. AB, while the other main B" regulatory subunits (hPR72, hPR70, mG5PR/B"γ, mPR59/B"δ1 and mPR59/B"δ2) were visualised as EGFP fusion proteins. Pictures were taken with a LSM-510 laser scanning confocal microscope.

Using GFP-fusion proteins, we determined the subcellular localisation of the hPR72, mPR59/B"δ1, mPR59/B"δ2, mG5PR/B"γ and hPR70 subunits in COS7 cells. Since the PR130 AB is suitable for direct immunofluorescence, we also evaluated the subcellular localisation of endogenous PR130 (Figure 9). As previously described [11], hPR72 is mainly present in the nucleus (N >> CP). PR130 is present in both the cytoplasm and the nucleus (N > CP). mPR59/B"δ1 (CP = N) and mPR59/B"δ2 (CP > N) appear both in cytoplasm and nucleus but are clearly less abundant in the nucleus than hPR72 and PR130. mG5PR/B"γ is more abundant in the nucleus than the cytoplasm (N > CP), corresponding to earlier published data [9], and additionally is highly abundant in the cell periphery. Surprisingly, unlike PR48 [20], hPR70 has a more pronounced cytoplasmic expression (CP >> N). This diversity in observed subcellular localisation patterns demonstrates that each B" subunit may direct the phosphatase to distinct sites and substrates within the cell.

### All B" subunits tested provoke a G1 arrest upon overexpression

**Figure 10:**
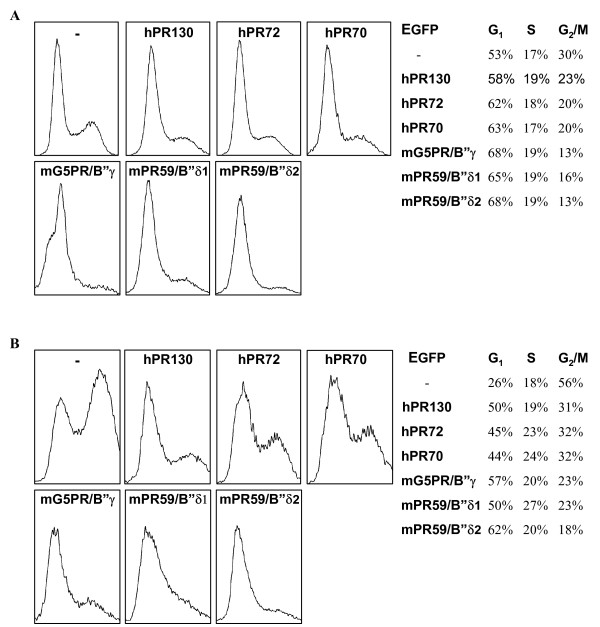
**Cell cycle profile of EGFP, hPR72-EGFP, hPR130-EGFP, hPR70-EGFP, mG5PR/B"γ-EGFP, mPR59/B"δ1-EGFP and mPR59/B"δ2-EGFP expressing HeLa cells**. 48 h after transfection with the different expression plasmids, HeLa cells were stained for DNA content (propidium iodide), and the cell cycle profile of EGFP-positive cells was measured. Panel A contains untreated cells whereas cells of panel B were treated with nocodazole (16 h, 1 mg/ml) prior to analysis. Quantification of the percentages of cells in G1, S and G2/M stages of the cell cycle are included. These are results from one typical experiment. Each condition was performed at least 4 times giving similar results but the outcome was highly dependent on the (over)expression level.

Several B" family members have been suggested to influence cell cycle progression [9-11,13,14,20]. Therefore we analysed the cell cycle profile of HeLa cells upon overexpression of several EGFP-B" fusion-proteins via FACS analysis. Compared to control cells (EGFP), overexpression of all EGFP-B" fusion proteins tested (hPR72, hPR130, hPR70, mG5PR/B"γ, mPR59/B"δ1 and mPR59/B"δ2) caused an increase in the G1 population of transfected cells (Figure 10A). This was even more pronounced in the presence of nocodazole, which arrests cycling cells in G2/M (Figure 10B). These results confirm and extend previously published effects of hPR72 [11] and mPR59/B"δ2 [10] overexpression on cell cycle progression.

## Discussion

The importance of the B-type subunits for PP2A regulation can hardly be overestimated as they restrict PP2A activity spatially and temporarily within living organisms, thereby determining PP2A substrate selectivity and function. Although the B-type subunits can be classified into three distinct gene families (PR55/B, PR61/B' and PR72/B"), their still increasing number, the sometimes confusing nomenclature and species-specific differences add to the complexity, especially for the non-specialist. In particular, for the PR72/B" subunit family the current situation may be confusing. With the aim to make this more clear and at the same time to provide a platform for the generation of B" knockout mice, we have presented here a comprehensive overview of the murine B" family members-in analogy to previous reports of the murine B' [25] and B [24] families.

In addition to the previously described murine-specific PR59 subunit [10] and the murine orthologues of PR130, PR72 and G5PR, we have identified two novel splice variants of mPR59 as members of the murine B" family. Based on the genomic organisation of the *Ppp2r3d *gene, we named the original isoform PR59/B"δ2 and the novel isoforms PR59/B"δ1 and PR59/B"δ3. In contrast to PR59/B"δ1 and δ2, no *in vivo *interaction of PR59/B"δ3 with PP2A could be observed. Consequently, PR59/B"δ3 can not be considered as a regulatory subunit of PP2A. In addition, other potential murine B" family members (mPR130/B"α3, mPR72/B"α4 and mPR59/B"δ4) were found to be present in the NCBI EST database. Although genomic evidence for these isoforms could be found, our attempts to confirm their existence at either the mRNA or protein level failed. This suggests that these isoforms are poorly expressed or might result from splicing errors. Nevertheless, we can not exclude the possibility that their expression is missed because of a tight regulation or restriction in space and/or time. Moreover, the existence of two B" isoforms (PR130/B"α3 and PR59/B"δ3) which do not interact with the PP2A core dimer, gives further support to the idea that B" subunits can exist- and be stable-independently of PP2A and that their function might extend beyond regulation of phosphatase activity as such (see [4] for a discussion). Even if so far only minute amounts of these atypical unexpected messages could be detected, specific conditions might lead to an upregulation and possible functional consequences.

To our surprise, no murine orthologues of human PR70 or PPP2R3L product could be found. These gene products arise from the *PPP2R3B/L *gene, and according to the genomic organisation of *PPP2R3B/L*, we propose to rename these two N-terminal splice variants PR70/B"β1 and PR70/B"β2 respectively (Table 2). Several arguments suggest that the murine *Ppp2r3d *gene (PR59) originates from a common ancestor of the human *PPP2R3B/L *gene (PR70), the *Canis familiaris PPP2R3B *gene (PR70) and the *Xenopus laevis LOC398610 *gene (PR70): (1) none of these organisms contain both the genes responsible for the expression of PR59 and PR70 isoforms; (2) these genes share the highest degree of homology amongst the B" members, both on the protein as well as on the genomic level; (3) from both genes, two B" regulatory subunits arise by using alternative first exons; and (4) human PR70/B"β1 can interact with and regulate the phosphorylation status of pRb while murine PR59/B"δ2 can associate with and regulate the phosphorylation status of p107, another member of the retinoblastoma family [10]. Therefore, they may share some functional similarities. Despite these similarities, we do not consider PR59 as the murine orthologue of PR70. This is based on (1) the rather low sequence homology between the specific N-terminal regions of the different PR59 and PR70 isoforms and (2) the ubiquitous expression of both PR59 regulatory subunits in contrast to the skeletal muscle and heart-specific expression of hPR70/B"β2[8]. For these reasons, we suggested to rename the PR59-encoding gene *Ppp2r3d*, instead of *Ppp2r3b*. Because of the diversity between mice and humans, it can be concluded that in contrast with the PP2A PR55/B and PR61/B' families, the PR72/B" family represents a less evolutionary conserved group of proteins. However, this does not mean that this family is not represented in lower species. PR72/B" members are also found in *Xenopus laevis *[7], *Drosophila melanogaster *[GenBank:NM_142585], *Caenorhabditis elegans *[GenBank:XM_001667031], various plants (*Arabidopsis thaliana *[GenBank:AF290025], *Oryza sativa *[GenBank:AC078829]), and some unicellular organisms (*Tetrahymena thermophila *[GenBank:XP_001015505], *Paramecium tetraurelia *[GenBank:XP_001347004]), but are manifestly absent in yeast.

**Table 2:** Proposed nomenclature of PP2A PR72/B" regulatory subunits in *Homo sapiens *and *Mus musculus*.

GENE SYMBOL				PROTEIN			
HUMAN		MOUSE		HUMAN		MOUSE	
name	alias	(new) name	alias	new name	alias	new name	alias

*PPP2R3A*	*PPP2R3*	*Ppp2r3a*	*3222402P14Rik*	**PR130/B"α1**	**PR130**	**PR130/B"α1**	**PR130**
				**PR72/B"α2**	**PR72**	**PR72/B"α2**	**PR72**
						PR130/B"α3	
						PR72/B"α4	

*PPP2R3B*	*NY-REN-8*	-	-	**PR70/B"β1**	PR70*	-	-
	*PPP2R3L*			**PR70/B"β2**	PPP2R3L product *	-	-

*PPP2R3C*	*C14orf10*	*Ppp2r3c*	*MGC55473*	**G5PR/B"γ**	**G5PR**	**G5PR/B"γ**	**G5PR**

-	-	*Ppp2r3d*	*AI118493*	-	-	**PR59/B"δ1**	
				-	-	**PR59/B"δ2**	**mPR59**
				-	-	PR59/B"δ3	
				-	-	PR59/B"δ4	

bold: main PR72/B" regulatory subunits

*: protein of which first a partial clone, PR48, was discovered

Unlike the PR55/B and PR61/B' family, the nomenclature of both the genes and proteins of the PR72/B" family members became very confusing. As mentioned earlier, we propose to change the murine PR72/B" gene names in such a way that the relationship with their human counterparts becomes clearer (Table 2). As for the PR72/B" proteins, until now, most of them have been named according to their molecular weight (with G5PR as an exception to this rule). In this report, we would like to propose a nomenclature which is in accordance with the corresponding gene name and the organisation of that gene, and is consistent with the nomenclature of the proteins of the PR55/B and PR61/B' families. For historical reasons we prefer to keep the original and commonly known PR130, PR72, PR70, PR59 and G5PR protein names combined with the B" indication and an additional specification (Greek letter) to denote the specific isoform. As such, B" proteins encoded by the *PPP2R3A *gene (PR130 and PR72) will get the 'α' extension, followed by a number to designate the specific splice variant; B" proteins encoded by the *PPP2R3B *gene (PR70) get the 'β' extension etc. For the comfort of the authors and readers, once in a manuscript the subunit is clearly defined, the short names B"α1, B"γ1 etc. can be used. In Table 2, a summary of the (old and new) murine and human B" nomenclature is given. If one intents to indicate the whole family, one can use PR72/B" as a common denominator since this makes an unequivocal distinction with the other B-type third subunits: PR55/B and PR61/B'.

We have also investigated the tissue distribution and developmental expression of all murine PP2A PR72/B" subunits on the RNA level. Taking together previously published data [6-8] and our northern blot analyses, it can be concluded that mPR130/B"α1, hPR130/B"α1, mPR59/B"δ1, mPR59/B"δ2, mG5PR/B"γ and XPR70/B"β1 are ubiquitously expressed, indicating that these subunits are involved in general cellular processes. In contrast, mPR72/B"α2, hPR72/B"α2, and hPR70/B"β2 are highly expressed in heart and skeletal muscle and barely detectable in other tissues, suggesting a specific role for these subunits in striated muscle tissues. In fact, all B" subunits seem to be highly expressed in heart, an observation which is given the presence of two EF-hands in their primary structures and their regulation by Ca^2+ ^ions, of particular interest.

Immunohistochemical analysis of longitudinal fibers of murine heart and skeletal muscle showed a striated pattern for mPR130/B"α1 and mPR72/B"α2. Counterstaining with iron hematoxylin revealed that they both colocalise with the A-band. In contrast to the I-band, the A-band does not only contain thin, actin-rich filaments but also thick, myosine-rich filaments. mPR130/B"α1 and mPR72/B"α2 staining is virtually absent in the smooth muscle of the bladder, suggesting that these subunits may play a specific role in striated muscle contraction only. Although mPR130/B"α1 and mPR72/B"α2 stain the nuclei in longitudinal as well as cross sections of heart and skeletal muscle, their nuclear localisation in these tissues is less pronounced compared to cultured fibroblast cell lines, probably due to their relatively high abundance in the contractile apparatus.

During embryogenesis no significant differences in expression from E7 until E17 could be observed for transcripts of mG5PR/B"γ, mPR59/B"δ1 and mPR59/B"δ2, indicating that the expression of these proteins is not regulated during early embryonic development. Meanwhile, mPR130/B"α1 and mPR72/B"α2 expression increases from E7 to E17. This might imply a role for these proteins during early embryogenesis. Alternatively, protein levels of these isoforms are simply building up for a more maximal expression during late development or after birth. In accordance with this increased expression during embryogenesis, depletion of XPR72 in *Xenopus *embryos caused several severe developmental defects (defects in somite formation, a short axis phenotype and lack of eye differentiation) [15]. Depletion of XPR130 resulted in milder developmental defects such as a disruption of somite organisation and an underdeveloped tail [16]. Together these data provide valuable information that will aid in the development of suitable strategies to generate future B" knockout mice, as well as in their phenotypic analysis.

At the cellular level, we have also determined the subcellular localisation of the main murine and human B" subunits via expression of EGFP-fusion proteins in COS7 cells (for PR72/B"α2, G5PR/B"γ, PR70/B"β1, PR59/B"δ1 and PR59/B"δ2) or via indirect immunofluorescence (for PR130/B"α1). A high diversity was observed: whereas PR130/B"α1, PR72/B"α2 and G5PR/B"γ are predominantly nuclear, PR59/B"δ1 is equally well expressed in the cytoplasm, and PR70/B"β1 and PR59/B"δ2 are predominantly expressed in the cytoplasm. The latter observation further extends the relationship and functional homology between these two isoforms. On the other hand, the cytoplasmic localisation of PR70/B"β1 is in contrast with the reported nuclear localisation of its truncated PR48 form [20], suggesting that nuclear import/export of PR70 isoforms may be a regulated event. Further, putative nuclear localisation signals are predicted in G5PR/B"γ (mono- and bipartite), PR130/B"α1 and PR72/B"α2 (monopartite), but not in PR59/B"δ1, PR59/B"δ2 or PR70/B"β1, whereas a nuclear export signal is predicted in the common part of PR59/B"δ1 and PR59/B"δ2. Although the functionality of these motifs remains to be determined, their putative occurrence/absence fits quite well with our experimental observations.

Upon overexpression, all B" subunits tested can induce a G1/S cell cycle arrest in HeLa cells, suggesting that this property may be independent of their subcellular localisation. The failure to proceed to S-phase may be related to the suggestion that many B" subunits play roles in DNA replication [9,10,13,14,20]. However, the mechanism of this induced cell cycle arrest may equally well be the result of a dominant negative effect, such as for instance a competition between 'free' B" [11] and trimeric B" for binding a substrate, or competition between B" and other B-type subunits for binding to PP2A_D_.

## Conclusion

In conclusion, the murine PR72/B" family exhibits an equal complexity and diversity as the PR55/B and PR61/B' families of PP2A subunits, with three genes encoding nine isoforms/splice variants, of which at least five are abundantly expressed and give rise to genuine PP2A subunits. An important difference with the PR55/B and PR61/B' families however, is the poorer relationship between human and murine genes, which might indicate that the B" family is evolutionary more divergent. Moreover, the diversity of PR72/B" members does not only originate from the number of isoforms, but extends to their specific tissue distribution, developmental expression and subcellular localisation. These differences add to the notion that no matter how similar their primary structures may be, different PP2A B-type isoforms likely display different *in vivo *functions. To explore these functions in mouse models remains an exciting challenge for the future.

## Methods

### Materials

[^35^S]-Methionine (2.5 mCi/ml), [^32^P]UTP, Protein G Sepharose and Glutathione Sepharose beads were obtained from Amersham Pharmacia Biotech. Restriction enzymes and DNA-modifying enzymes were purchased from Fermentas. *Pwo *proofreading polymerase (used in all PCR reactions) was from Roche Molecular Biochemicals. DNA oligonucleotides were purchased from Sigma-Genosys.

### Bioinformatics

To search for the murine B" homologues in public databases we used the Nucleotide BLAST programs at NCBI . For the alignment of proteins and generation of a phylogenetic tree, we used the multalin program . Percentages of similarity and identity between the amino acid sequence of two proteins were calculated using EMBOSS Pairwise alignment algorithm . Data regarding the localisation of genes on the murine chromosomes were obtained using the ENSEMBLE Contig View , while the corresponding regions in human chromosomes were found using the ENSEMBLE Synteny View. Putative nuclear localisation signals have been predicted using the PSORT program , whereas putative nuclear export signals were predicted using the NetNES 1.1 Server .

### Antibodies

Anti-GST monoclonal antibody was purchased from Sigma. Anti PP2A_C _and anti-PR65 monoclonal antibodies were generously supplied by Dr. S. Dilworth (Imperial College, London, UK). Anti-PR72_N _pept. antibody was a generous gift of Dr. B. Hemmings (FMI, Basel, CH). Anti-PR72_rec. _antibody has been previously described [11]. For the generation of the anti- PR130 _N rec. _antibody, BL21-pLys(RP) *E. coli *cells were transformed with PR130 AA1-664 in pET15b and induced with 0.2 mM IPTG overnight at 16°C. The inclusion bodies were purified [11], solubilised in 7 M guanidinium hydrochloride, and dialysed against 200 mM Tris. HCl pH 8.2 containing 0.5 M NaCl. The resulting protein solution was used to immunise rabbits (Unité d'Hormonologie, Marloie, Belgium). The antiserum was taken after 5 boosts with the antigen and used at 1/2000 for Western blotting and 1/100 for immunofluorescence. Before immunisation a sample of the preimmune serum was taken as a negative control.

### RNA isolation and RT-PCR

Total RNA from NIH 3T3 cells and murine heart tissue was isolated using GenElute™ Mammalian Total RNA Kit (Sigma) and reverse transcribed using primer E_3 _(5'-CTG TGC GCC CAG GAA CTG CGC-3') for the mPR59 isoforms and primers E_5 _(5'-CCT TGC AGT CCT CTT CCA CCA GC-3'), E_7 _(5'-GAT TCT GTC AGG GCT CTA GCA GG-3') and E_9 _(5'-GGA TGC TGT GTA GAG ATG CTC TAG-3') for the mPR72/130 isoforms. The following primers were used to amplify the different mPR59 isoforms: B"δ1 (5'-CTC TGC CAT CAG TCT CTG CCC-3'), Bδ2 (5'-CAG GCC ACA CCC ACG GAT TCG-3'), B"δ3/4 (5'-CCA CAG GGT CTT CAG CCA CGC-3'), E_1 _(5'-GCC CTG CCC ACT CTG ACT ATG-3') and E_2 _(5'-GAC GAC CTG GAG CCT CTG TGA-3'). The primers used to amplify the different mPR72/130 isoforms are B_130 _(5'-ATG GCA GCA ACT TAC AGA CTT GTG-3'), B_72 _(5'-ATG ATC AAG GAA ACG TCC TTG CGA AG-3'), E_4 _(5'-CTG TTG CTT GGG CAG CCT GAC C-3'), E_6 _(5'-AGG TTC AGC TCC AAG AAG GGT GA-3') and E_8 _(5'-CTT CAG TCA GTG GAT GAA GAG TAG-3'). The approximate location of the various primers within the genes is indicated in Figure 3.

### *In vitro *transcription-translation

[^35^S]-Methionine-labelled proteins were obtained from pBluescript vectors containing the coding regions of the proteins, using the TNT-coupled rabbit reticulocyte lysate system (Promega) with the appropriate RNA polymerase (T3/T7).

### Immunoprecipitation of tissue extracts

Wild-type mice were anaesthetised with an intraperitoneal injection of pentobarbital (Nembutal, CEVA), transcardially perfused with ice-cold saline (NaCl 0.9%, B Braun) and dissected tissues were freeze clamped in liquid nitrogen. Tissues were weighed and homogenised on ice within a threefold volume excess of homogenisation buffer (25 mM Tris pH 7.6, 150 mM NaCl, 1 mM EDTA and 1 mM EGTA) containing protease inhibitors (Complete Protease Inhibitor Cocktail, Roche) using a douncer with 20–30 strokes. The homogenates were centrifuged for 10' at 13,000 g and the supernatant was collected. After preclearing with protein G-Sepharose (Amersham Biosciences), tissue lysates were incubated overnight at 4°C with anti-PR72_N rec._, anti-PR130_N rec. _or anti-PR72_N pept._. After addition of 40 μl of protein G-Sepharose for 1 h, the immune complexes were washed 4 times with 1 ml TBS + 0.1% Nonidet P-40 before they were dissolved in Laemmli sample buffer. Bound proteins were separated by SDS/PAGE and transferred to nitrocellulose membranes. Individual proteins were detected with the specified antibodies, revealed by horseradish peroxidase-linked secondary antibodies (DAKO) and developed using the ECL kit (Amersham Biosciences).

### GST-pull down

Monkey COS7 cells were transfected (FuGENE 6, Roche) with pGMEX-T1, hPR72/B"α2-pGMEX, hPR130/B"α1-pGMEX, hPR70/B"β1-pGMEX, mPR59/B"δ1-pGMEX, mPR59/B"δ2-pGMEX, mPR59/B"δ3-pGMEX and mG5PR/B"γ-pGMEX in a 10-cm dish. 48 h after transfection, cells were rinsed with phosphate-buffered saline (PBS), lysed in 200 μl NET buffer (50 mM Tris pH 7.4, 150 mM NaCl, 15 mM EDTA and 1% NP-40) and centrifugated for 10' at 13,000 g. Cell lysates were incubated at 4°C for 1 h with NENT_200 _buffer (20 mM Tris-HCl pH 7.4, 1 mM EDTA, 0.1% Nonidet P-40, 25% glycerol, 200 mM NaCl) containing 1 mg/ml bovine serum albumin and 25 μl glutathione-Sepharose beads (Amersham Biosciences) on a rotating wheel. The beads were washed 2 times with 1 ml of NENT_300 _(NENT with 300 mM NaCl) containing 1 mg/ml bovine serum albumin and 2 times with 1 ml of NENT_300_. Bound proteins were eluted by addition of 20 μl of Laemmli sample buffer and boiling. The eluted proteins were analysed by SDS-PAGE and Western blotting.

### Construction of isoform-specific RNA probes

To generate isoform-specific PCR products, we used following primers: 5'-ATG ATC AAG GAA ACG TCC TTG CGA AG-3' and 5' GAA GAG GCT GAA GTC ATT TCA G-3', generating mPR72/B"α2 nt 1–120; 5'-ATG GCA GCA ACT TAC AGA CTT GTG-3' and 5'-GAA TAC CTG TAA CTT AAA GGA C-3', resulting in mPR130/B"α1 nt 1–330; 5'-atg gac tgg a aa gac gtg ctt c-3' and 5'-gcc aaa ctc ctt cat aca gat-3', producing mG5PR/B"γnt 1–450; 5'-ATG GCG CCG CTG ACG CCG CGG-3' and 5'-GAA CGA CGG GAC CCA GGA CG-3', producing mPR59/B"δ1 nt 1–243; and 5'-AGG CCA CAC CCA CGG ATT CG-3' and GTT TCC GTC GCG CTT CCA GAA G-3', resulting in mPR59/B"δ2 54 nt 5'UTR + nt 1–120 of coding region. We subcloned these PCR products into the pBluescript II SK-vector (Stratagene). The constructs were used to generate RNA probes. The RNA probes were transcribed and labeled with [^32^P]UTP using Ambion's Strippable RNA probe synthesis and removal kit.

### Northern blotting

A Mouse Tissue MTN^® ^Blot containing poly(A^+^) RNA from eight murine tissues and a Mouse Embryo MTN^® ^Blot containing poly(A^+^) RNA from murine embryos at days 7, 11, 15 and 17 were obtained from Clontech Laboratories. The membranes were hybridised at 64°C using Ambion's Ultrahyb Ultrasensitive hybridisation buffer and washed with Northern Max Low and High Stringency Buffers from Ambion. They were exposed and analysed using ImageQuant (Molecular Dynamics). Probe removal was done using Ambion's Strippable RNA probe synthesis and removal kit. For quantification we used the ImageQuant program from Molecular Dynamics. Absolute values of the different tissues and embryonic stages were first divided by the absolute β-actin values of the corresponding tissue/embryonic stages. These values where then compared with the value of the most intense band on the tissue blot, which was given a value of 100%.

### Subcellular localisation

PCR fragments of hPR72/B"α2, hPR70/B"β1, mG5PR/B"γ, mPR59/B"δ1 and mPR59/B"δ2 were cloned into the *Sma*I restriction site of pEGFP-C1 (Clontech). 1 μg of each plasmid was transfected into COS7 cells grown on a glass coverslip in a 12-well dish. 24 h after transfection, cells were washed in PBS and fixed in PBS containing 4% paraformaldehyde for 10 min. After 3 washes with PBS, cells were mounted in Prolong gold antifade reagent with DAPI (Invitrogen). For immunofluorescence staining of endogenous PR130, COS7 cells grown on a glass coverslip were rinsed with PBS and fixed in PBS with 4% paraformaldehyde for 10 min. Subsequently, cells were incubated with PBS containing 1% bovine serum albumin (BSA, Sigma) for 30 min and then for 45 min with the PR130_N rec. _primary antibodies, followed by incubation with Alexa Fluor 488 donkey anti-rabbit secondary antibody (Invitrogen) in PBS with 1% BSA. Finally, the cells were washed 3 times in PBS, once in water and mounted in Prolong gold antifade reagent with DAPI (Invitrogen). All slides were examined with a LSM-510 laser scanning confocal microscope (Zeiss, Jena, Germany), using a Plan-Neofluar 40×/1.6 Oil DIC objective.

### FACS Analysis

Asynchronously growing HeLa cells were transfected with pEGFP-C1 or the GFP fusion plasmids of the different B" subunits in two 10-cm dishes per plasmid. If nocodazole (1 μg/ml) was used, it was added at this point for another 16 h before FACS analysis. 48 h after transfection, cells were trypsinised, washed in PBS and fixed for 5 min in 4% paraformaldehyde at room temperature. After washing, the cell pellet was incubated in 0.5 ml of PBS containing 100 μg/ml propidium iodide (Fluka) and 0.1% RNase for at least 1 h at room temperature. The samples were analysed with a Beckman Instruments Coulter Epics XL flow cytometer (Analis) on FL1 (for EGFP) and FL3 (for propidium iodide) using standard procedures, and the System II™ software (Analis) and WinMDI Version 2.8 software for quantification.

### Immunohistochemistry

Wild-type mice were anaesthetised with pentobarbital (Nembutal, CEVA) and transcardially perfused with PBS containing 4% paraformaldehyde. Organs were removed, postfixed overnight at 4°C and paraffin embedded. Sections (7 μM) were mounted on silan coated glass slides (Menzel gläser). For antigen retrieval, dewaxed and rehydrated tissue sections were boiled in 10 mM citric acid (pH 6.0) for 10 min. After cooling the slides, endogenous peroxidase was removed with 0.03% H_2_O_2 _in methanol for 20 min and transferred to PBS solution. Subsequently, sections were encircled with a water-repellent PAP-pen (Zymed) and rinsed with PBS. After blocking with PBS containing 1% BSA for 45 min, sections were immunoreacted for 1 h at RT with primary antibodies, washed 3 times with PBS containing 0.05% Tween (PBS-T), incubated with biotinylated goat anti-Rabbit secondary antibody (Vector Labs) at RT for 30 min, and washed three times with PBS-T. After incubation for 30 min with the VECTASTAIN ABC Systems (Vector Labs), sections were washed 3 times in PBS-T, incubated with diaminobenzidine tetrahydrochloride (1 tablet dissolved in 10 ml 50 mM Tris pH 7.6, MP Biomedicals), washed three times in water and incubated with hematoxylin (Sigma) or iron hematoxylin (Sigma) for 2 min. Next, sections were washed in tap water for 5', dehydrated and mounted using Depex. Tissue sections were examined using a light microscope equipped with a digital camera (DC200, Leica Microsystems).

## List of abbreviations

AB (antibody), ASBD (A subunit binding domain), CG-NAP (centrosome and Golgi localized PKN-associated protein), DARPP-32 (dopamine- and cAMP-regulated phosphoprotein of 32 kDa), FeH (iron hematoxylin), hPR130 (human PR130), PKA (protein kinase A), mPR59 (murine PR59), PKN (novel protein kinase), PP1 (protein phosphatase 1), PP2A (protein phosphatase 2A), PP2A_D _(PP2A heterodimer), PP2A_TX _(PP2A trimer with x as third subunit), Nkd (Naked Cuticle), PP5 (protein phosphatase 5), pRb (retinoblastoma protein)

## Authors' contributions

KZ has contributed to the design and execution of most of the presented experiments, the analysis and interpretation of the obtained data and writing of the manuscript. JVL has contributed to the design and realisation of immunohistochemistry experiments and critical assessment of the paper. JG has contributed to the design of experiments, the analysis and interpretation of the data and critical assessment of the manuscript. VJ has contributed to the design and execution of experiments, the analysis and interpretation of the data and writing of the manuscript. All authors read and approved the final manuscript.
